# Supplementary material for: Metabolic and transcriptomic reprogramming during contact inhibition-induced quiescence is mediated by YAP-dependent and YAP-independent mechanisms
Source: Nat Commun. 2024 Aug 8;15:6777. doi: 10.1038/s41467-024-51117-y (PMC11310444; doi:10.1038/s41467-024-51117-y)
Supplement: Supplementary file 1 — Supplementary Information [file 41467_2024_51117_MOESM1_ESM.pdf]

Supplementary Information

Metabolic and transcriptomic reprogramming during contact inhibition-induced quiescence is mediated by YAP-dependent and YAP-independent mechanisms

The file includes Supplementary Table 1 and Supplementary Figures 1-11

Soeun Kang[1], Maciek R. Antoniewicz[2], Nissim Hay[1,3,4]

[1]Department of Biochemistry and Molecular Genetics, College of Medicine, University of Illinois at Chicago, Chicago, IL 60607, USA

[2]Chemical Engineering Department, University of Michigan, Ann Arbor, MI 48109, USA

[3]Research and Development Section, Jesse Brown VA Medical Center, Chicago, IL 60612, USA

[4]Lead Contact

*Correspondence: nhay@uic.edu (N. H.)

Supplementary Table 1

Key resources table

**REAGENT or RESOURCE**	**SOURCE**	**IDENTIFIER**
**Plasmids**		
**Plasmid Backbone**	**Insert**	**Ref**
pBabe hygro	EV	Addgene #1765
pBabe hygro	YAP WT	This paper
pBabe hygro	YAP 5SA	This paper
LentiCRISPRv2 Blast	EV	Addgene #83480
LentiCRISPRv2 Blast	sgCdh1	This paper
LentiCRISPRv2 Blast	sgYAP1	This paper
LentiCRISPRv2 Blast	sgTead1	This paper
LentiCRISPRv2 Hygro	sgSlc25a11	This paper
pPB Blast	hME1-Myc	Vector builder (Vector ID: VB201218-1018fnx)
pPB Neo	hME1-Flag	Vector builder (Vector ID: VB201218-1275dct)
pPB Neo	iNap1	Vector builder (Vector ID: VB210324-1055kjr)
pLX304	pMOS023: Peredox NADH/NAD+ sensor (cytosolic)	Addgene #163060
pC1	mitoRexYFP	Addgene #60246
pLV	EV	Navdeep Chandel (Northwestern University)
pLV	Cyto-LbNOX	Navdeep Chandel (Northwestern University)
pLV	Mito-LbNOX	Navdeep Chandel (Northwestern University)
pLVX	tetONE-zeo-MPC2-P2A-T2A-MPC1	Jared Rutter (University of Utah)
**Antibodies**		
**Target**	**Company**	**Catalogue #**
MYC TAG	Cell Signaling Technology	2276S
Flag TAG	Cell Signaling Technology	2368S
Mouse monoclonal anti-FLAG	Sigma-Aldrich	F1804; RRID:AB_262044
HA TAG	Sigma-Aldrich	H-9658
Vinculin	Sigma	V9131; RRID:AB_477629
Tubulin	Sigma	7-9062
HK1	Invitrogen	MA5-15675

HK2	Cell Signaling Technology	2867S
PFK1	abcam	ab97443
ALDOA	Cell Signaling Technology	8060S
PKM1	Cell Signaling Technology	7067T
PKM2	R&D Systems	MAB72442
LDHA	Invitrogen	MA5-17246
$p27^{Kip1}$	BD Biosciences	610241
MPC1	Cell Signaling Technology	14462S
MPC2	Cell Signaling Technology	46141S
IDH2	Cell Signaling Technology	56439S
DLST	Cell Signaling Technology	11954S
SDHA	Cell Signaling Technology	11998S
Fumarase	Cell Signaling Technology	4567T
CS	Cell Signaling Technology	14309T
MDH2	Invitrogen	PA5-21760
YAP1	Cell Signaling Technology	12395
Phosphorylated YAP1 (S127)	Cell Signaling Technology	4911S
TEAD1	Cell Signaling Technology	12292S
PCK1	Cell Signaling Technology	12940S
MDH1	Invitrogen	PA5-97965
ME1	Invitrogen	MA5-23524
ME1	Proteintech	16619-1-AP
SIRT6	Invitrogen	MA5-24768
ACAT1	Cell Signaling Technology	44276S
Acetylated Lysine	Cell Signaling Technology	9441S
SLC25A11	Invitrogen	PA5-101673
SLC25A13	Proteintech	10789-1-AP
ALDH18A1 (P5CS)	Invitrogen	PA5-52546
PYCR1	Cell Signaling Technology	47935
TOMM20	Invitrogen	PA5-52843
COL1A1	NOVUS BIO	NBP1-30054
COL2A1	NOVUS BIO	NBP1-77795
COL3A1	Proteintech	22734-1-AP
COL14A1	Invitrogen	PA5-49916
COL23A1	Invitrogen	MA5-24188
Goat anti-Mouse IgG (H+L) Cross-Adsorbed Secondary Antibody, Alexa Fluor™ 488	Invitrogen	A11001
Goat anti-Rabbit IgG (H+L) Cross-Adsorbed Secondary Antibody, Cyanine3	Invitrogen	A10520
**Bacterial and Virus Strains**		
Stbl3 bacteria	Thermo Fisher Scientific	C737303

DH5α competent cells	NEB	C2987H
FastDigest BsmBI & 10X FD buffer	Thermo Fisher Scientific	FD0454
FastAP	Thermo Fisher Scientific	EF0651
T4 PNK	NEB	M0201S
Quick Ligase	NEB	M2200S
**Chemicals, Peptides, and Recombinant Proteins**		
**Reagents**	**Source**	**Catalogue #**
Fetal bovine serum	Gemini	100-106
Dialyzed fetal bovine serum	Gemini	100-108
Penicillin/streptomycin	Corning	30-002-CI
DMEM / High Glucose	Corning	10-017-CV
DMEM w/o Glucose, Sodium pyruvate	Gibco	1966-025
DMEM w/o Glucose, Glutamine, Serine, Glycine, Sodium pyruvate	USBiological	D9802-01
OptiMEM	Thermo Fisher Scientific	31985070
0.25% Trypsin 2.21 mM EDTA	Corning	25-053-CI
DMSO	Fisher bioreagents	BP231-100
HPLC Grade Water	Sigma	7732-18-5
HPLC Grade Methanol	Alfa Aesar	22909
HPLC Grade Chloroform	Alfa Aesar	22920
Seahorse XF base medium	Agilent	102353-100
Lipofectamine 2000	Thermo Fisher Scientific	11668019
FuGENE	Promega	E5911
1,2-$^{13}C_2$ glucose	Cambridge Isotopes Laboratory	CLM-504-PK
U^{13}C glucose	Cambridge Isotopes Laboratory	CLM-1396
U^{13}C glutamine	Cambridge Isotopes Laboratory	CLM-1822
MOX™ Reagent	Thermo Scientific	TS45950
MTBSTFA	Cerilliant	M-108-5X1ML
N-Methyl-N-(trimethylsilyl)trifluoroacetamide	Sigma	394866-10X1ML
Glutamine	Sigma	G8540
Glucose	Sigma	G7021
Polybrene	Sigma	H9268
Puromycin	Gibco	A11138-03
Blasticidin	Gibco	A11139-03
Virapower	Thermo Fisher Scientific	K497500
VSV-G	Addgene	8454
10X RIPA buffer	CST	9806
Protease inhibitor tablet	Thermo Fisher Scientific	A32963

Phosphatase inhibitor tablet	Thermo Fisher Scientific	A32957
Protein Assay Dye	Bio-Rad	5000006
Ponceau	Sigma-Aldrich	P7170
Difco™ skim milk	BD	232100
Immobilon®-FL PVDF Membrane	Millipore	IPFL00010
Tween	Thermo Fisher Scientific	3005
BSA	Sigma-Aldrich	A2153-100G
Hoechst 33342, trihydrochloride, trihydrate	Thermo Fisher Scientific	H3569
Propidium iodide	Sigma-Aldrich	P4170-25MG
PureLink™ RNase A	Invitrogen	12091-021
0.45μm filter	Millipore	SLHV004SL
0.22μm filter	Millipore	SLGVV255F
Poly-L-lysine	Sigma	P4707
Metformin hydrochloride	Sigma-Aldrich	PHR1084
FCCP (carbonyl cyanide 4-(trifluoromethoxy)phenyl hydrazone)	Sigma-Aldrich	C2920
Rotenone	Sigma-Aldrich	R8875
Oligomycin A	Sigma-Aldrich	75351
Antimycin A	Sigma-Aldrich	A8674
Tetramethylrhodamine, Ethyl Ester, Perchlorate (TMRE)	Invitrogen	T669
A/G agarose beads	Santa Cruz	sc-2003
Ms anti-Bromodeoxyuridine Clone Bu20a	Dako	M0744
**Critical commercial assays**		
GeneJET RNA purification kit	Thermo Fisher Scientific	K0732
GeneJET Plasmid Miniprep kit	Thermo Fisher Scientific	K0503
NAD/NADH-Glo Assay kit	Promega	G9072
Dual-Luciferase® Reporter Assay System	Promega	E1980
iScript cDNA Synthesis Kit	Bio-Rad	1708891
iQ SYBR Green Supermix	Bio-Rad	1708882
Direct-zol RNA Microprep Kit	ZYMO RESEARCH	R2060
Pierce™ Rapid Gold BCA Protein Assay Kit	Thermo Scientific	A53225

QIAquick PCR Purification Kit	QIAGEN	28104
**Experimental models: Cell lines**		
**Name**	**From**	**Ref**
Mouse embryonic fibroblast (MEFs)	In house	[15,16]
Primary MEFs	In house	[15,16]
NIH 3T3	ATCC	CRL-1658
BJ	ATCC	CRL-2522
293FT	ThermoFisher	R70007
Phoenix-AMPHO	ATCC	CRL-3213
CHO-K1	ATCC	CCL-61
**Oligonucleotides – qPCR primers**		
**qPCR Oligo**	**Sequence**	**Ref**
HK1	For : GCTCAGAAAAGGGGGATTTC<br>Rev : GATGTTCTCTGGGGTGTCGT	Primer-3
HK2	For : TGATCGCCTGCTTATTCACGG<br>Rev : AACCGCCTAGAAATCTCCAGA	[54]
PFK1	For : CCATGTTGTGGGTGTCTGAG<br>Rev : ACAGGCTGAGTCTGGAGCAT	Primer-3
ALDOA	For : CTGAGCGACCACCATGTCTA<br>Rev : GGCAATCTCCTCATTGGAAA	Primer-3
PKM1/2	For : CGATCTGTGGAGATGCTGAA<br>Rev : AATGGGATCAGATGCAAAGC	Primer-3
LDHA	For : AGGCTCCCCAGAACAAGATT<br>Rev : TCTCGCCCTTGAGTTTGTCT	Primer-3
$P27^{Kip1}$	For : AACTAACCCGGGACTTGGAG<br>Rev : CCAGGGGCTTATGATTCTGA	Primer-3
Cyclophilin E	For : TTCACAAACCACAATGGCACAGGG<br>Rev : TGCCGTCCAGCCAATCTGTCTTAT	Lester Lau (UIC)
MPC1	For : TTGAAAGAGACAGTGCTCCAG<br>Rev : GTTTAGGGACTCTCGGCTATTC	this paper
MPC2	For : CTGCAGAGAGAAAGTGACGAG<br>Rev : TGCCGGGTGGTTGTAAAG	this paper
NDUFS2	For : TTTCGGGAGCTGTCATGTACC<br>Rev : TGGTCACCGCTTTTTCCTTCA	[55]
IDH2	For : GGCTGTCAAGTGTGCCACAATC<br>Rev : TTGGCTCTCTGAAGACGGTTCC	https://www.origene.com
DLST	For : CCACCTGTGTGGAGGTATTG<br>Rev : GTGCTTTCTGGAAGAGGTAGAG	this paper
SDHA	For : CAGACCTGTTATCGACAAGACC	this paper

	Rev : GTTTCATCAGTAGGAGCGGATAG	
CCN1	For : GAGGCTTCCTGTCTTTGGCAC<br>Rev : ACTCTGGGTTGTCATTGGTAAC	56
Cdc25c	For :<br>GGTCCAAACAAAGGAAGATATTACAC<br>Rev : GTCCTCAAGGTCAGCAGAAG	this paper
Cdc20	For : GTGTCCTTAGCTTGTCTGGAAA<br>Rev : GTTGAAGCAGTGAGTGCAAATC	this paper
Aurka	For : GTCTTGGTGACTGAGCAGATT<br>Rev : GTTATTGAGCCGGGACACAG	this paper
Aurkb	For : TGAGATTGGGCGTCCTTTG<br>Rev : CGCCTGGATTTCGATCTCTC	this paper
Plk1	For : GATCTCAGACGCAGACACAAA<br>Rev : ATACAAAGTCGCTGTCCTCAAA	this paper
Cenps	For : AATGAAGAGATTGCCCAGCTTA<br>Rev : GCTGCTTCCAGATGAGATGAA	this paper
Bub1	For : GCTGAGTACAACAGTGACC<br>Rev : CCTCATTGTGAATTCCTGTCT	this paper
Mcm2	For :<br>CGAGGAGGATGAAGAGATGATCG<br>Rev : GCTCCTTGAAGACGTTGTGG	this paper
Col1a1	For : CGATGGATTCCCGTTCGAGT<br>Rev : CGATCTCGTTGGATCCCTGG	57
Col1a2	For : GCAGGTTCACCTACTCTGTCCT<br>Rev : CTTGCCCCATTCATTTGTCT	58
Col3a1	For : CTGGAGAACCTGGTGCAAAT<br>Rev : CCTCGGAAGCCACTAGGAC	58
Col4a3	For : TGTGGATGCACGGTGTGTT<br>Rev : GTTCTCTTCACGGTGTGCTTGA	57
Col5a1	For :<br>AAGCGTGGGAAACTGCTCTCCTAT<br>Rev :<br>AGCAGTTGTAGGTGACGTTCTGGT	59
Col5a2	For : GAAAGGCTGGTGATCAAGGT<br>Rev : TTTCTCCCCGAGGTCCTAAT	58
Col6a1	For : GATGAGGGTGAAGTGGGAGA<br>Rev : CAGCACGAAGAGGATGTCAA	hc9_2023_04_20_hirsova_hep4-22-0898r1_sdc2.pdf (lww.com)
Col6a3	For : CATGACTCGCCAACA<br>Rev : GGATGGCATTGAAGTTGGAT	58
Col8a1	For : TCTGCCACCTCAAATCCCTCCTCA<br>Rev : TCTCCGCGCAAACTGGCTAACG	60
Col8a2	For : GAGCGACGCGGAGTTCTG<br>Rev : CCCCCTGCATGGCGTCTGTG	60
Col11a1	For : GACCAGAAGACACACTGAAAGCA	61

	Rev : TCCATGCCATCTGAGTAGTCAAGA	
Col14a1	For : GACAGAGTGAACGACAGGAAAG<br>Rev : TGGCGCCTAGGGAAGATTA	this paper
Col23a1	For : CCAAGGGCAAGATGGAGTT<br>Rev : CATCTTTCCCAGTGTCACCA	this paper
Col5a3	For : TCTCCAGCAGATCCTCACA<br>Rev : CTCCGGCAGCTTCTCATC	this paper
**Oligonucleotides**		
**Cloning Oligo**	**Sequence**	**Ref**
sgCdh1	For : CACCGGAACGTGTCCGGCTCTCGAG<br>Rev : AAACCTCGAGAGCCGGACACGTTCC	CRISPOR.tefor.net
sgYAP1	For : CACCGCCAGAGACAACGCCACTGGCT<br>Rev : AAACAGCCAGTGGCGTTGTCTCTGGC	[62]
sgTead1	For : CACCGCCGATTGACAACGACGCGGA<br>Rev : AAACTCCGCGTCGTTGTCAATCGGC	CRISPOR.tefor.net
sgSlc25a11	For : CACCGGCTGAAAGTCGTCCGCTATG<br>Rev : AAACCATAGCGGACGACTTTCAGCC	CRISPOR.tefor.net
**Equipment and Software**		
**Machine**	**Company**	**Model**
Chemiluminescent western blot developer	Azure	cSeries Imaging Systems c400
Near-infrared fluorescence western blot developer	Licor	Odyssey CLx
qPCR machine	Bio-Rad	CFX96
Flow-cytometer	Beckman Coulter	CytoFLEX S
Live cell imaging	Nexcelom Bioscience	Celigo Imaging Cytometer
GC-MS	Agilent	7890A/5975C
Plate reader	BioTek	CYTATION1
Biochemistry Analyzer	Yellow Springs Instruments (YSI)	YSI-2700 Select
Seahorse	Seahorse Bioscience	XFe96 Extracellular Flux Analyzer
Confocal microscope	Zeiss	LSM 700 &<br>LSM 900
**Software**	**Company**	**Prod #**

ZEN Blue	Zeiss	https://www.zeiss.com/microscopy/en/products/software/zeiss-zen-lite
Image Studio Lite	Licor	https://www.licor.com/bio/image-studio-lite/
Prism 9	GraphPad	https://www.graphpad.com/
FlowJo 10.1	FlowJo LLC	https://www.flowjo.com/
Matlab R2016b (9.7.0)	MathWorks	https://uk.mathworks.com
R (4.2.2)		https://cran.r-project.org/bin/windows/base/
Wave	Agilent XF Software	https://www.agilent.com/en/product/cell-analysis/real-time-cell-metabolic-analysis/xf-software/seahorse-wave-desktop-software-740897
Wave	Agilent XF Software	https://www.agilent.com/en/product/cell-analysis/real-time-cell-metabolic-analysis/xf-software/seahorse-wave-desktop-software-740897

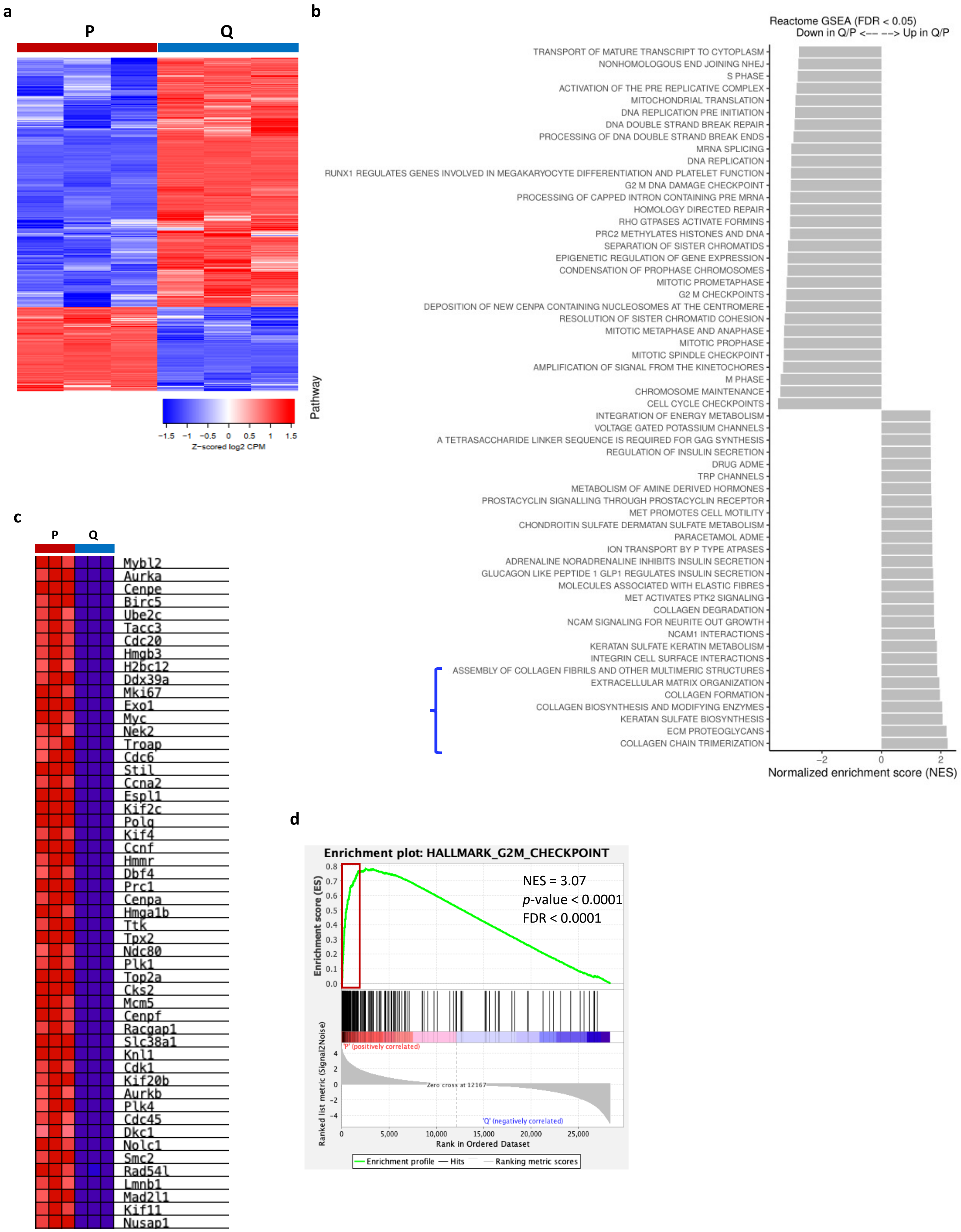
a
P
Q
−1.5 −1 −0.5 0 0.5 1 1.5
Z−scored log2 CPM
b
Reactome GSEA (FDR < 0.05)
Down in Q/P <–– ––> Up in Q/P
TRANSPORT OF MATURE TRANSCRIPT TO CYTOPLASM
NONHOMOLOGOUS END JOINING NHEJ
S PHASE
ACTIVATION OF THE PRE REPLICATIVE COMPLEX
MITOCHONDRIAL TRANSLATION
DNA REPLICATION PRE INITIATION
DNA DOUBLE STRAND BREAK REPAIR
PROCESSING OF DNA DOUBLE STRAND BREAK ENDS
MRNA SPLICING
DNA REPLICATION
RUNX1 REGULATES GENES INVOLVED IN MEGAKARYOCYTE DIFFERENTIATION AND PLATELET FUNCTION
G2 M DNA DAMAGE CHECKPOINT
PROCESSING OF CAPPED INTRON CONTAINING PRE MRNA
HOMOLOGY DIRECTED REPAIR
RHO GTPASES ACTIVATE FORMINS
PRC2 METHYLATES HISTONES AND DNA
SEPARATION OF SISTER CHROMATIDS
EPIGENETIC REGULATION OF GENE EXPRESSION
CONDENSATION OF PROPHASE CHROMOSOMES
MITOTIC PROMETAPHASE
G2 M CHECKPOINTS
DEPOSITION OF NEW CENPA CONTAINING NUCLEOSOMES AT THE CENTROMERE
RESOLUTION OF SISTER CHROMATID COHESION
MITOTIC METAPHASE AND ANAPHASE
MITOTIC PROPHASE
MITOTIC SPINDLE CHECKPOINT
AMPLIFICATION OF SIGNAL FROM THE KINETOCHORES
M PHASE
CHROMOSOME MAINTENANCE
CELL CYCLE CHECKPOINTS
INTEGRATION OF ENERGY METABOLISM
VOLTAGE GATED POTASSIUM CHANNELS
A TETRASACCHARIDE LINKER SEQUENCE IS REQUIRED FOR GAG SYNTHESIS
REGULATION OF INSULIN SECRETION
DRUG ADME
TRP CHANNELS
METABOLISM OF AMINE DERIVED HORMONES
PROSTACYCLIN SIGNALLING THROUGH PROSTACYCLIN RECEPTOR
MET PROMOTES CELL MOTILITY
CHONDROITIN SULFATE DERMATAN SULFATE METABOLISM
PARACETAMOL ADME
ION TRANSPORT BY P TYPE ATPASES
ADRENALINE NORADRENALINE INHIBITS INSULIN SECRETION
GLUCAGON LIKE PEPTIDE 1 GLP1 REGULATES INSULIN SECRETION
MOLECULES ASSOCIATED WITH ELASTIC FIBRES
MET ACTIVATES PTK2 SIGNALING
COLLAGEN DEGRADATION
NCAM SIGNALING FOR NEURITE OUT GROWTH
NCAM1 INTERACTIONS
KERATAN SULFATE KERATIN METABOLISM
INTEGRIN CELL SURFACE INTERACTIONS
ASSEMBLY OF COLLAGEN FIBRILS AND OTHER MULTIMERIC STRUCTURES
EXTRACELLULAR MATRIX ORGANIZATION
COLLAGEN FORMATION
COLLAGEN BIOSYNTHESIS AND MODIFYING ENZYMES
KERATAN SULFATE BIOSYNTHESIS
ECM PROTEOGLYCANS
COLLAGEN CHAIN TRIMERIZATION
Pathway
−2 0 2
Normalized enrichment score (NES)
c
P
Q
Mybl2
Aurka
Cenpe
Birc5
Ube2c
Tacc3
Cdc20
Hmgb3
H2bc12
Ddx39a
Mki67
Exo1
Myc
Nek2
Troap
Cdc6
Stil
Ccna2
Espl1
Kif2c
Polq
Kif4
Ccnf
Hmmr
Dbf4
Prc1
Cenpa
Hmga1b
Ttk
Tpx2
Ndc80
Plk1
Top2a
Cks2
Mcm5
Cenpf
Racgap1
Slc38a1
Knl1
Cdk1
Kif20b
Aurkb
Plk4
Cdc45
Dkc1
Nolc1
Smc2
Rad54l
Lmnb1
Mad2l1
Kif11
Nusap1
d
Enrichment plot: HALLMARK_G2M_CHECKPOINT
NES = 3.07
p-value < 0.0001
FDR < 0.0001
Enrichment score (ES)
0.0 0.1 0.2 0.3 0.4 0.5 0.6 0.7 0.8
'P' (positively correlated)
Zero cross at 12167
'Q' (negatively correlated)
Ranked list metric (Signal2Noise)
4 2 0 -2 -4
0 5,000 10,000 15,000 20,000 25,000
Rank in Ordered Dataset
Enrichment profile — Hits — Ranking metric scores

Supplementary figure 1

a. Heatmap of the differentially expressed genes between P and Q MEFs as determined by RNA sequencing. Data represent values from three independent experiments, and Z scored log2-fold change values are color-coded as indicated.

b. GSEA identified the differentially expressed Reactome pathways in P and Q MEFs. The top 30 down- and 28 upregulated pathways selected by statistical significance (there were only 28 upregulated pathways with $FDR < 0.05$ in Q/P). Top 7 most upregulated pathways in Q cells are marked with blue curly bracket.

c. Heatmap of the genes in the G_2-M checkpoint pathway by GSEA from RNA sequencing of P and Q MEFs. Expression values are represented as colors and range from red (high expression), pink (moderate), light blue (low) to dark blue (lowest expression).

d. Enrichment of genes in the G_2-M checkpoint pathway by GSEA from RNA sequencing of P and Q MEFs. The normalized enrichment score (NES), *p*-value and false discovery rate (FDR) are shown.

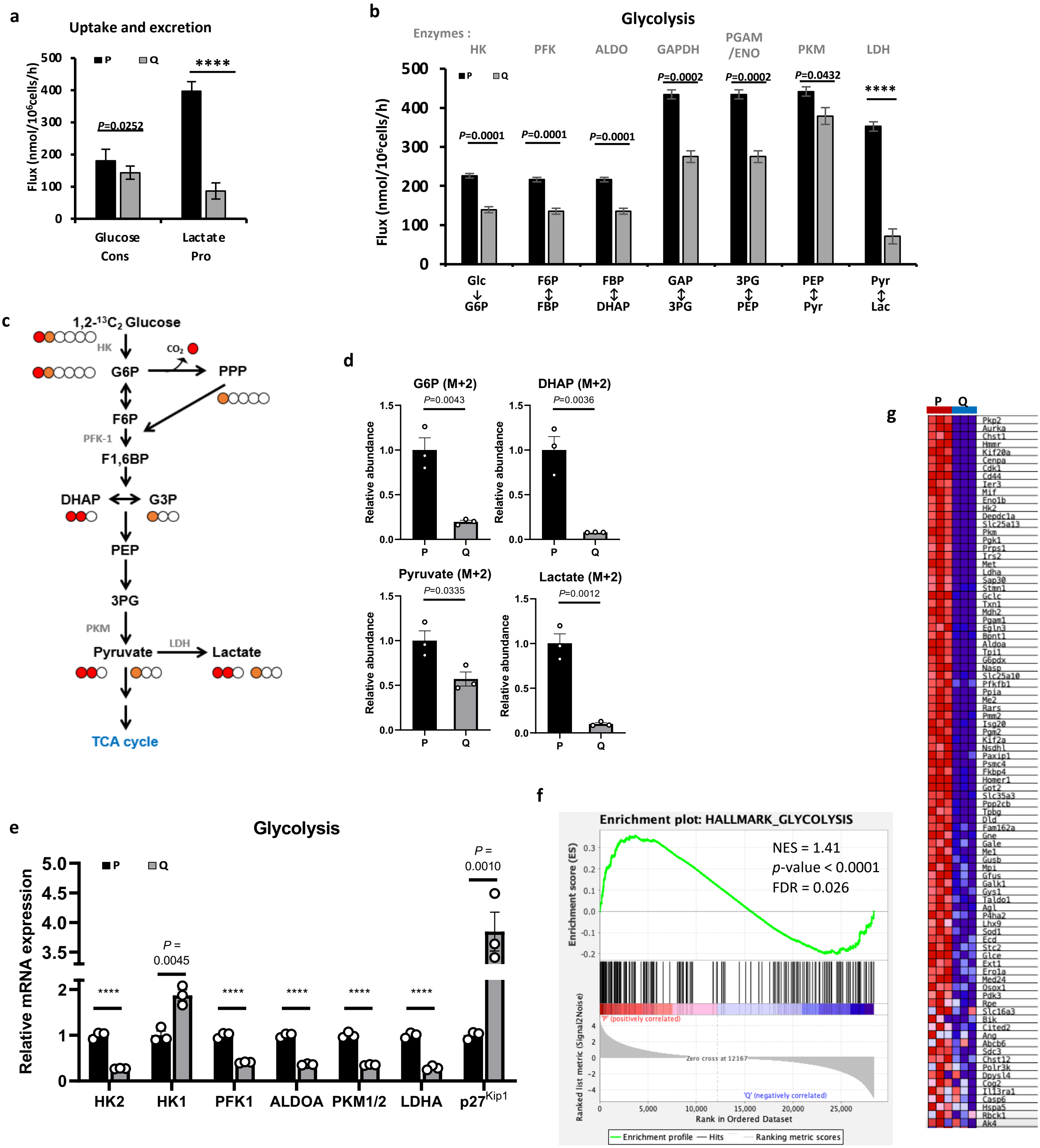

a
Uptake and excretion
Flux (nmol/10^6cells/h)
P Q
P=0.0252
****
Glucose Cons
Lactate Pro
b
Glycolysis
Enzymes :
HK PFK ALDO GAPDH PGAM /ENO PKM LDH
Flux (nmol/10^6cells/h)
P Q
P=0.0001
P=0.0001
P=0.0001
P=0.0002
P=0.0002
P=0.0432
****
Glc → G6P
F6P ↕ FBP
FBP ↕ DHAP
GAP ↕ 3PG
3PG ↕ PEP
PEP ↕ Pyr
Pyr ↕ Lac
c
1,2-13C2 Glucose
HK
CO2
G6P
PPP
F6P
PFK-1
F1,6BP
DHAP
G3P
PEP
3PG
PKM
LDH
Pyruvate
Lactate
TCA cycle
d
G6P (M+2)
P=0.0043
DHAP (M+2)
P=0.0036
Pyruvate (M+2)
P=0.0335
Lactate (M+2)
P=0.0012
Relative abundance
P Q
e
Glycolysis
Relative mRNA expression
P Q
****
P = 0.0045
P = 0.0010
HK2 HK1 PFK1 ALDOA PKM1/2 LDHA p27Kip1
f
Enrichment plot: HALLMARK_GLYCOLYSIS
NES = 1.41
p-value < 0.0001
FDR = 0.026
Enrichment score (ES)
Ranked list metric (Signal2Noise)
'P' (positively correlated)
Zero cross at 12167
'Q' (negatively correlated)
Rank in Ordered Dataset
Enrichment profile — Hits Ranking metric scores
g
P Q
Pkp2
Aurka
Chst1
Hmmr
Kif20a
Cenpa
Cdk1
Cd44
Ier3
Mif
Eno1b
Hk2
Depdc1a
Slc25a13
Pkm
Pgk1
Prps1
Irs2
Met
Ldha
Sap30
Stmn1
Gclc
Txn1
Mdh2
Pgam1
Egln3
Bpnt1
Aldoa
Tpi1
G6pdx
Nasp
Slc25a10
Pfkfb1
Ppia
Me2
Rars
Pmm2
Isg20
Pgm2
Kif2a
Nsdhl
Paxip1
Psmc4
Fkbp4
Homer1
Got2
Slc35a3
Ppp2cb
Tpbg
Dld
Fam162a
Gne
Galе
Me1
Gusb
Mpi
Gfus
Galk1
Gys1
Taldo1
Agl
P4ha2
Lhx9
Sod1
Ecd
Stc2
Glce
Ext1
Ero1a
Med24
Osox1
Pdk3
Rpe
Slc16a3
Blk
Cited2
Ang
Abcb6
Sdc3
Chst12
Polr3k
Dpysl4
Cog2
Il13ra1
Casp6
Hspa5
Rbck1
Ak4

Supplementary figure 2

a. Comparison of the quantified key extracellular metabolic fluxes (estimated flux ± SD) in P and Q MEFs. The flux rates of glucose uptake and lactate excretion are plotted.

b. Comparison of key intracellular glycolytic fluxes (estimated flux ± SD) in P and Q MEFs. The black bars represent P cells, and the gray bars represent Q cells.

c. Simplified schematic of the steps in glycolysis and the pentose phosphate pathway (PPP), showing ^{13}C labeling patterns with [1,2-$^{13}C_2$]-glucose tracing. Red fills indicate ^{13}C-labeled carbons. Abbreviations: G6P, glucose-6-phosphate; F6P, fructose-6-phosphate; F1,6BP, fructose 1,6-bisphosphate; DHAP, dihydroxyacetone phosphate; G3P, glyceraldehyde-3-phosphate; PEP, phosphoenolpyruvate; 3PG, 3-phosphoglycerate.

d. Relative abundances of intracellular 2x^{13}C (M+2) glycolytic metabolites (G6P, DHAP, pyruvate, and lactate) after 25 mM [1,2-$^{13}C_2$]-glucose labeling for 24 h. The AUC was normalized to the levels of protein and internal standard. Means ± SEMs (n = 3) are shown.

e. Relative mRNA expression of glycolytic genes in P and Q MEFs. Data are normalized to the average value of P cells and are the mean ± SEM of 3 independent experiments.

f. Enrichment of genes in glycolysis by GSEA from RNA sequencing of P and Q MEFs. The normalized enrichment score (NES), *p*-value and false discovery rate (FDR) are shown.

g. Heatmap of the genes in glycolysis by GSEA from RNA sequencing of P and Q MEFs. Expression values are represented as colors and range from red (high expression), pink (moderate), light blue (low) to dark blue (lowest expression).

Means and SEMs are shown, unless otherwise indicated. Individual *P* values by unpaired two-tailed *t*-test are indicated in each figure, unless otherwise indicated. Otherwise, asterisks indicate the *P* value; ns *P* ≥0.05, * *P* <0.05, ** *P* <0.01, *** *P* <0.001, **** *P* <0.0001.

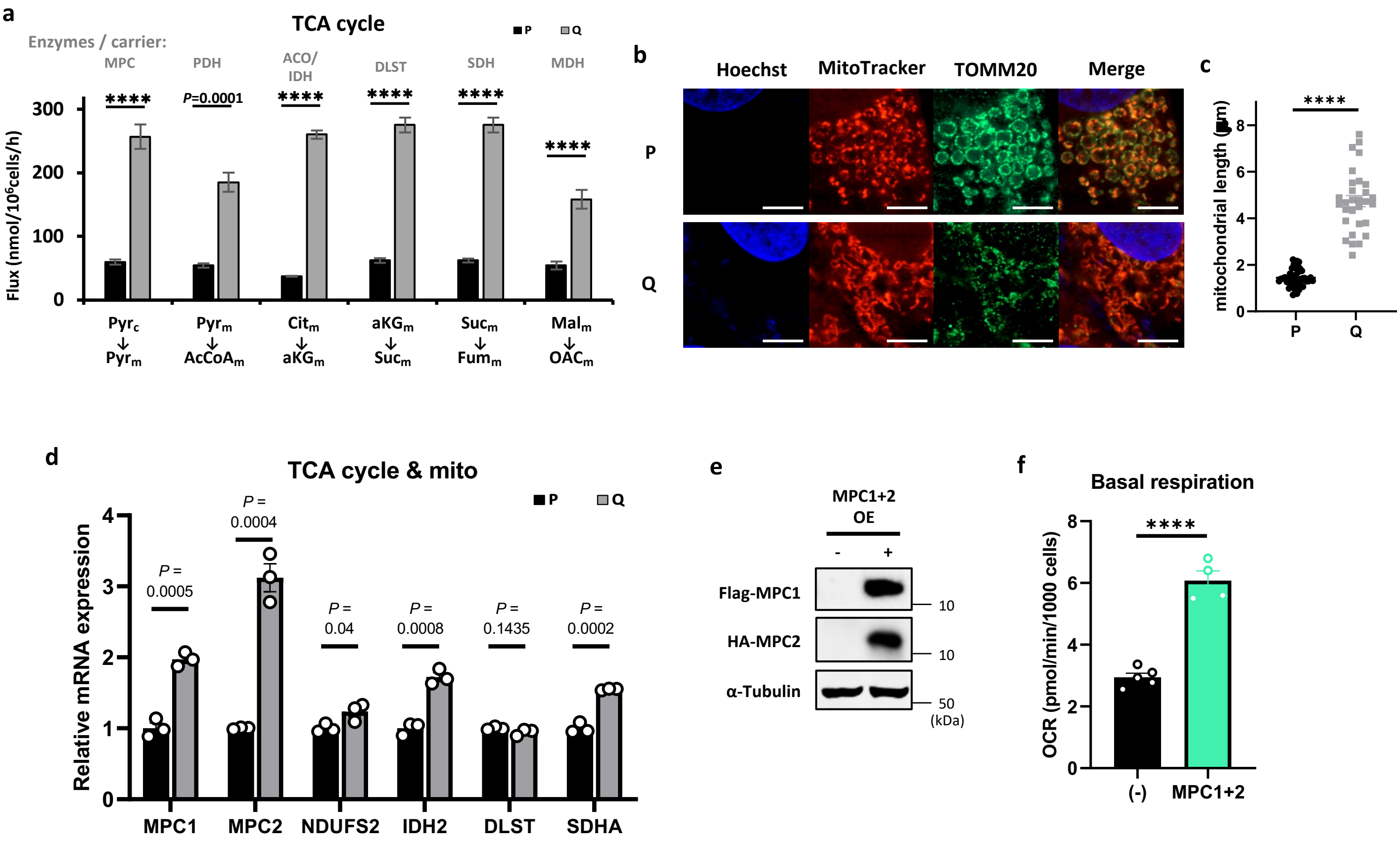

Supplementary figure 3

a. Comparison of key intracellular TCA cycle fluxes (estimated flux ± SD) in P and Q MEFs. The black bars represent P cells, and the gray bars represent Q cells.

b. Representative confocal images of MitoTracker (red), TOMM20 (green) and Hoechst 33342 (blue) immunostaining in P and Q MEFs. Scale bars: 5 µm.

c. Quantification of mitochondrial length. n=46 and 30 for P and Q cells, respectively.

d. Relative mRNA expression of MPC1-2, TCA cycle genes, and p27^{kip1} in P and Q MEFs. Data are normalized to the average value of P cells and are the mean ± SEM of 3 independent experiments.

e. Immunoblots of MPC1-2 in control and MPC1 and 2 overexpressing MEFs. MPC1 and 2 are stably expressed in the tet-on system, which were treated with doxycycline (0.2 µg/ml) for 5 days. α-Tubulin was monitored as a loading control. Flag and HA tags are used to target MPC1 and MPC2 respectively. Each immunoblot is representative of three independent experiments. Abbreviations: MPC1-2, mitochondrial pyruvate carrier 1-2.

f. Basal respiration measured by oxygen consumption rate (OCR) of control (-) and MPC1 and MPC2 overexpressing cells. Means ± SEMs (n ≥ 4) are shown.

Means and SEMs are shown, unless otherwise indicated. Individual *P* values by unpaired two-tailed *t*-test are indicated in each figure, unless otherwise indicated. Otherwise, asterisks indicate the *P* value; ns *P* ≥0.05, * *P* <0.05, ** *P* <0.01, *** *P* <0.001, **** *P* <0.0001.

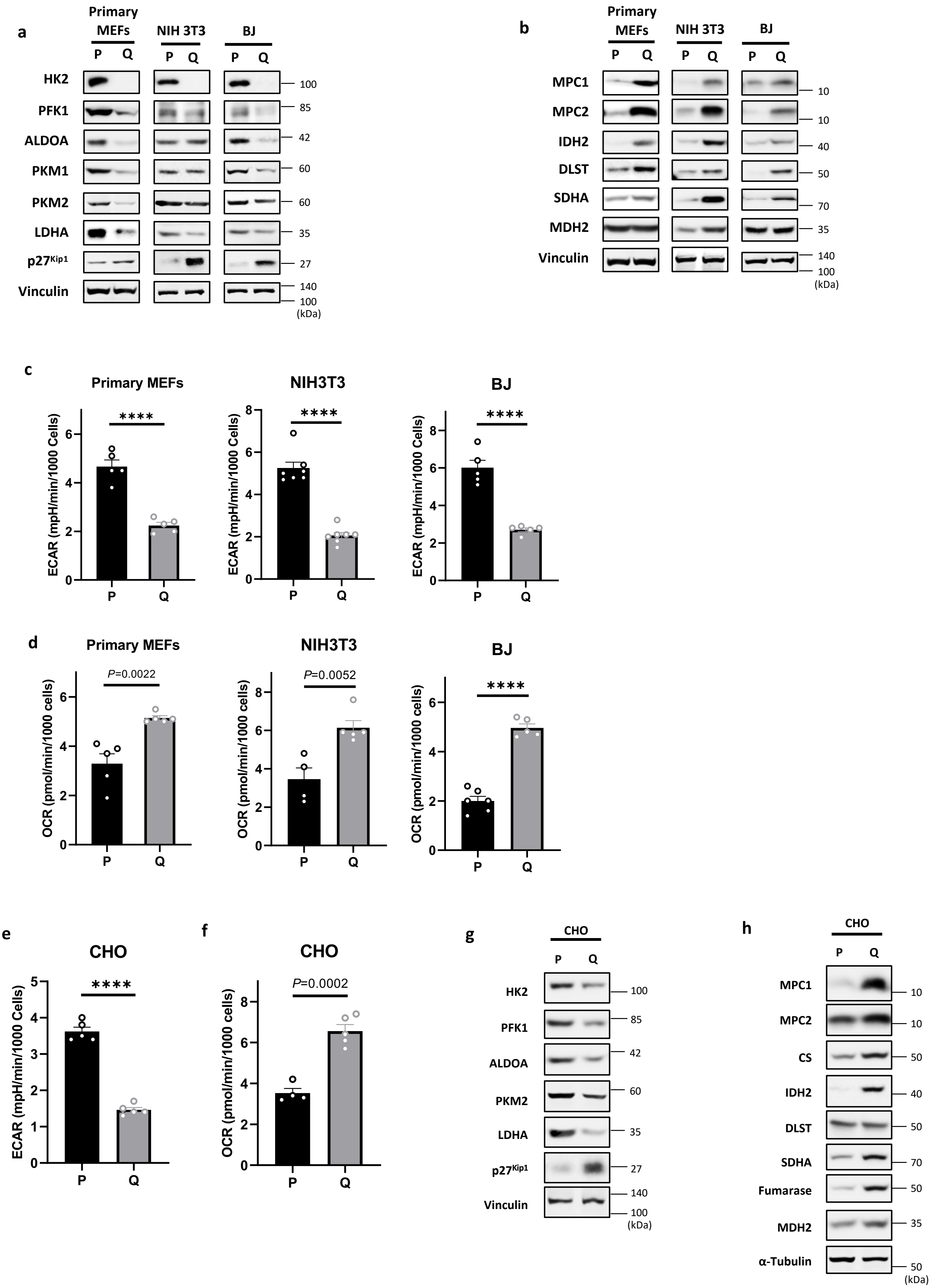
a
Primary MEFs
NIH 3T3
BJ
P Q P Q P Q
HK2
PFK1
ALDOA
PKM1
PKM2
LDHA
p27Kip1
Vinculin
100
85
42
60
60
35
27
140
100
(kDa)
b
Primary MEFs
NIH 3T3
BJ
P Q P Q P Q
MPC1
MPC2
IDH2
DLST
SDHA
MDH2
Vinculin
10
10
40
50
70
35
140
100
(kDa)
c
Primary MEFs
NIH3T3
BJ
ECAR (mpH/min/1000 Cells)
****
P Q
d
Primary MEFs
NIH3T3
BJ
OCR (pmol/min/1000 cells)
P=0.0022
P=0.0052
****
P Q
e
CHO
ECAR (mpH/min/1000 Cells)
****
P Q
f
CHO
OCR (pmol/min/1000 cells)
P=0.0002
P Q
g
CHO
P Q
HK2
PFK1
ALDOA
PKM2
LDHA
p27Kip1
Vinculin
100
85
42
60
35
27
140
100
(kDa)
h
CHO
P Q
MPC1
MPC2
CS
IDH2
DLST
SDHA
Fumarase
MDH2
α-Tubulin
10
10
50
40
50
70
50
35
50
(kDa)

Supplementary figure 4

a. Immunoblots of glycolytic enzymes in P and Q primary MEFs, NIH3T3, and BJ cells. Vinculin was monitored as a loading control. The immunoblots are representative of three independent experiments. Abbreviations: HK, hexokinase; PFK1, phosphofructokinase 1; ALDOA, aldolase A; PKM, pyruvate kinase M; LDHA, lactate dehydrogenase A.

b. Immunoblots of mitochondrial pyruvate carriers and TCA cycle enzymes in primary MEFs, NIH3T3, and BJ cells. Vinculin was monitored as a loading control. Each immunoblot is representative of three independent experiments. Abbreviations: MPC1-2, mitochondrial pyruvate carrier 1-2; IDH2, isocitrate dehydrogenase 2; DLST, dihydrolipoamide S-succinyltransferase; SDHA, succinate dehydrogenase; MDH2, malate dehydrogenase 2.

c. Glycolytic function monitored by the extracellular acidification rate (ECAR) in P and Q NIH3T3 cells. Means ± SEMs ($n \geq 4$) are shown.

d. Basal respiration measured by oxygen consumption rate (OCR) of P and primary MEFs, NIH3T3, and BJ cells. Means ± SEMs ($n \geq 4$) are shown.

e. Glycolytic function monitored by the extracellular acidification rate (ECAR) in P and Q CHO-K1 cells. Means ± SEMs ($n \geq 4$) are shown.

f. Basal respiration measured by oxygen consumption rate (OCR) of P and Q CHO-K1 cells. Means ± SEMs ($n \geq 4$) are shown.

g. Immunoblots of glycolytic enzymes in P and Q CHO-K1 cells. Vinculin was monitored as a loading control. The immunoblots are representative of three independent experiments. Abbreviations: HK, hexokinase; PFK1, phosphofructokinase 1; ALDOA, aldolase A; PKM, pyruvate kinase M; LDHA, lactate dehydrogenase A.

h. Immunoblots of mitochondrial pyruvate carriers and TCA cycle enzymes CHO-K1 cells. Vinculin was monitored as a loading control. Each immunoblot is representative of three independent experiments. Abbreviations: MPC1-2, mitochondrial pyruvate carrier 1-2; CS, citrate synthase; IDH2, isocitrate dehydrogenase 2; DLST, dihydrolipoamide S-succinyltransferase; SDHA, succinate dehydrogenase; MDH2, malate dehydrogenase 2.

Means and SEMs are shown, unless otherwise indicated. Individual *P* values by unpaired two-tailed *t*-test are indicated in each figure, unless otherwise indicated. Otherwise, asterisks indicate the *P* value; ns $P \geq 0.05$, * $P < 0.05$, ** $P < 0.01$, *** $P < 0.001$, **** $P < 0.0001$.

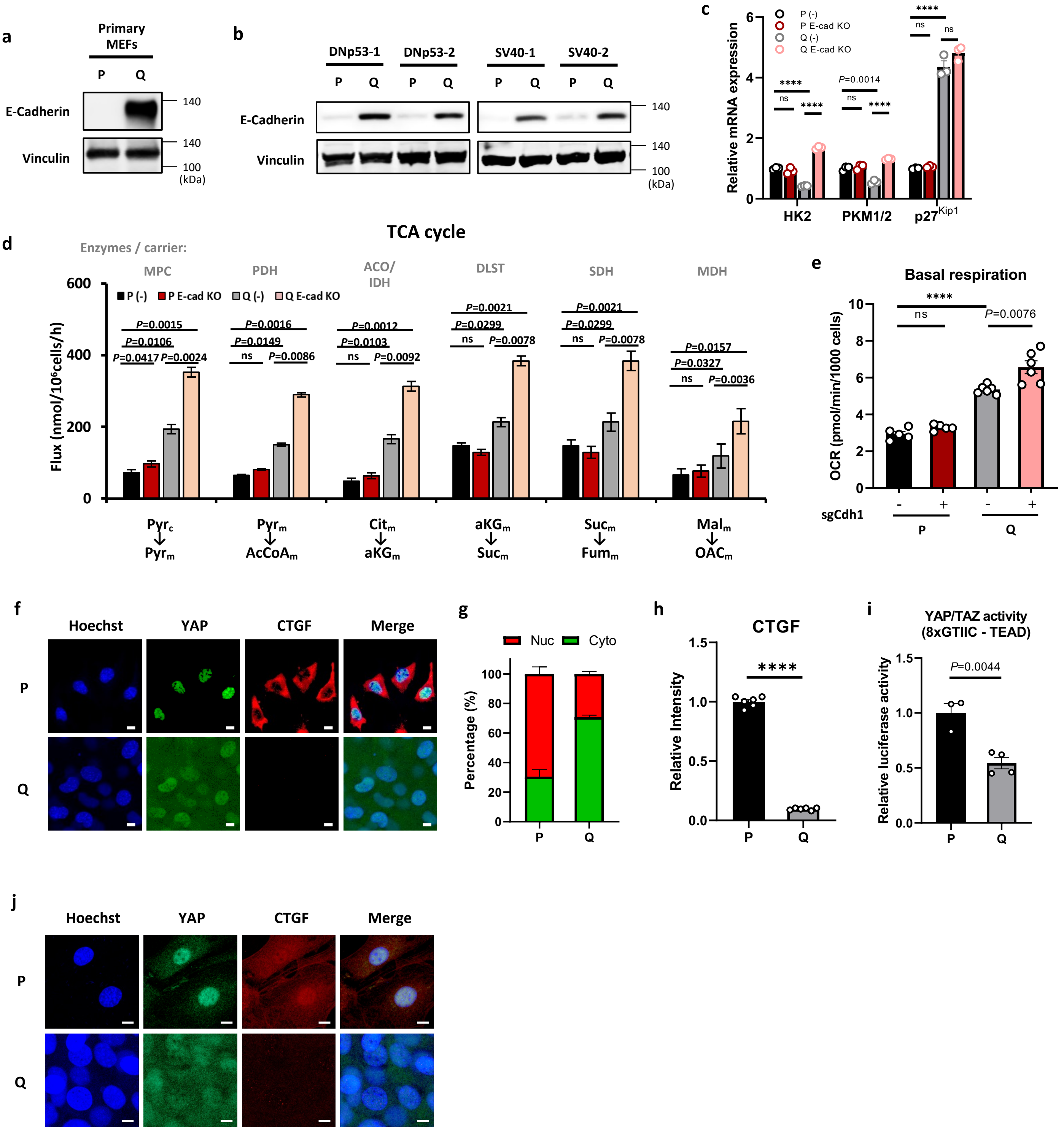
a
Primary MEFs
P
Q
E-Cadherin
Vinculin
140
140
100
(kDa)
b
DNp53-1
DNp53-2
SV40-1
SV40-2
P
Q
E-Cadherin
Vinculin
140
140
100
(kDa)
c
Relative mRNA expression
P (-)
P E-cad KO
Q (-)
Q E-cad KO
****
ns
****
P=0.0014
HK2
PKM1/2
p27Kip1
d
TCA cycle
Enzymes / carrier:
MPC
PDH
ACO/IDH
DLST
SDH
MDH
Flux (nmol/10⁶cells/h)
P (-)
P E-cad KO
Q (-)
Q E-cad KO
P=0.0015
P=0.0106
P=0.0417
P=0.0024
P=0.0016
P=0.0149
P=0.0086
P=0.0012
P=0.0103
P=0.0092
P=0.0021
P=0.0299
P=0.0078
P=0.0021
P=0.0299
P=0.0078
P=0.0157
P=0.0327
P=0.0036
ns
Pyrc → Pyrm
Pyrm → AcCoAm
Citm → aKGm
aKGm → Sucm
Sucm → Fumm
Malm → OACm
e
Basal respiration
OCR (pmol/min/1000 cells)
****
ns
P=0.0076
sgCdh1
P
Q
f
Hoechst
YAP
CTGF
Merge
P
Q
g
Nuc
Cyto
Percentage (%)
P
Q
h
CTGF
Relative Intensity
****
P
Q
i
YAP/TAZ activity (8xGTIIC - TEAD)
Relative luciferase activity
P=0.0044
P
Q
j
Hoechst
YAP
CTGF
Merge
P
Q

Supplementary figure 5

a. Immunoblots of E-Cadherin in primary P and Q MEFs. Each immunoblot is representative of three independent experiments, and vinculin was used as a loading control.

b. Immunoblots of E-Cadherin in P and Q MEFs immortalized with either dominant-negative p53 or SV40 large T. Each immunoblot is representative of three independent experiments, and vinculin was used as a loading control.

c. Relative mRNA expression of HK2, PKM1/2 and $p27^{Kip1}$ in control or E-cadherin KO MEFs in the P and Q states. Means ± SEMs (n = 3) are shown.

d. Comparison of key intracellular TCA cycle fluxes (estimated flux ± SD) in control or E-cadherin KO MEFs in the P and Q states.

e. Basal respiration measured by oxygen consumption rate (OCR) of control or E-cadherin KO MEFs in the P and Q states. Values are the mean ± SEM of 5~6 independent experiments.

f. Localization and expression analysis of YAP (green) and CTGF (red) in P and Q MEFs by immunofluorescence**.** Hoechst 33342 (blue) was used to show the nuclei, and the scale bar size represents 10 µm. Each experiment included observation of at least 10 randomly selected fields (400× magnification).

g. The quantified percentage (%) of nuclear (red) and cytosolic (green) YAP immunofluorescence is indicated, and the data represent the mean ± SEM (n=10). The statistical significance of the differences was determined by two-way ANOVA; P <0.0001.

h. The quantified intensity of CTGF immunofluorescence is indicated, and the data represent the mean ± SEM (n=6).

i. Luciferase assays with the 8×GTIIC-Lux reporter in control or E-cadherin KO MEFs in the P and Q states. Data are normalized to the average value of P cells and are presented as the mean ± SEM of 3 to 5 biologically independent samples.

j. Representative confocal images of YAP (green), CTGF (red) and Hoechst 33342 (blue) immunostaining in P and Q primary MEFs. Scale bars: 10 µm.

Means and SEMs are shown, unless otherwise indicated. Individual *P* values by unpaired two-tailed *t*-test are indicated in each figure, unless otherwise indicated. Otherwise, asterisks indicate the *P* value; ns *P* ≥0.05, * *P* <0.05, ** *P* <0.01, *** *P* <0.001, **** *P* <0.0001.

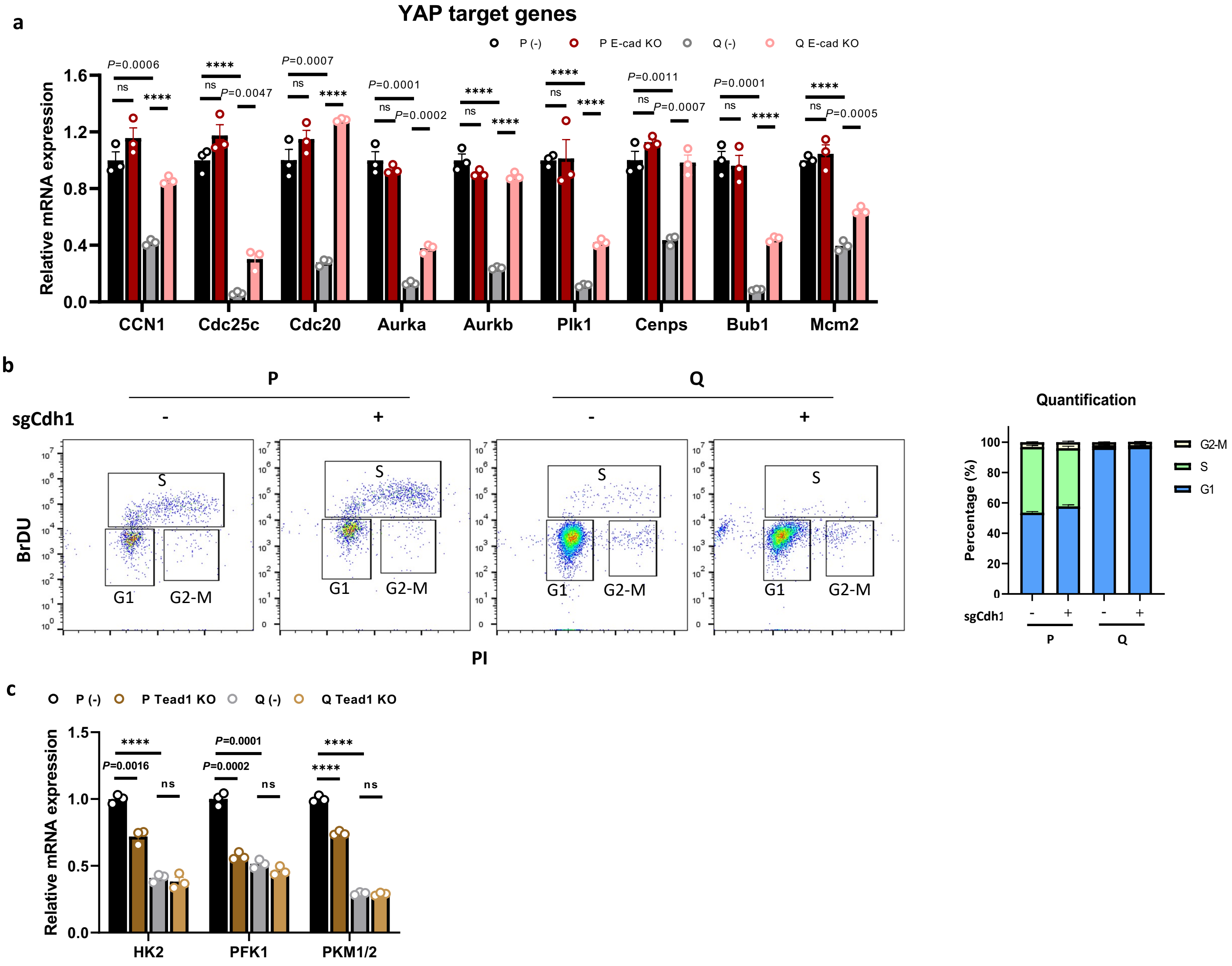

Supplementary figure 6

a. Relative mRNA expression of YAP target genes in control or E-cadherin KO MEFs in the P and Q states. Data are normalized to the average value of P cells and are the mean ± SEM of 3 independent experiments.

b. Cytometric analysis of BrdU incorporation and DNA content by PI staining in control or E-cadhe rin KO MEFs in the P and Q states. The box gates on dot plots represent G_1, S and G_2-M phases. The graph shows the quantification of cells in each phase of the cell cycle: cells arrested in G_1 phase (BrdU negative; 2N DNA content), in S phase (BrdU positive) and in G_2-M phase (BrdU n egative; 4N DNA content). The statistical significance of the differences was determined using two-way ANOVA; P <0.0001.

c. Relative mRNA expression of HK2, PFK1 and PKM1/2 in control or TEAD1 KO P and Q MEFs. M eans ± SEMs (n = 3) are shown.

Means and SEMs are shown, unless otherwise indicated. Individual *P* values by unpaired two-tailed *t*-test are indicated in each figure, unless otherwise indicated. Otherwise, asterisks indicate the *P* value; ns *P* ≥0.05, * *P* <0.05, ** *P* <0.01, *** *P* <0.001, **** *P* <0.0001.

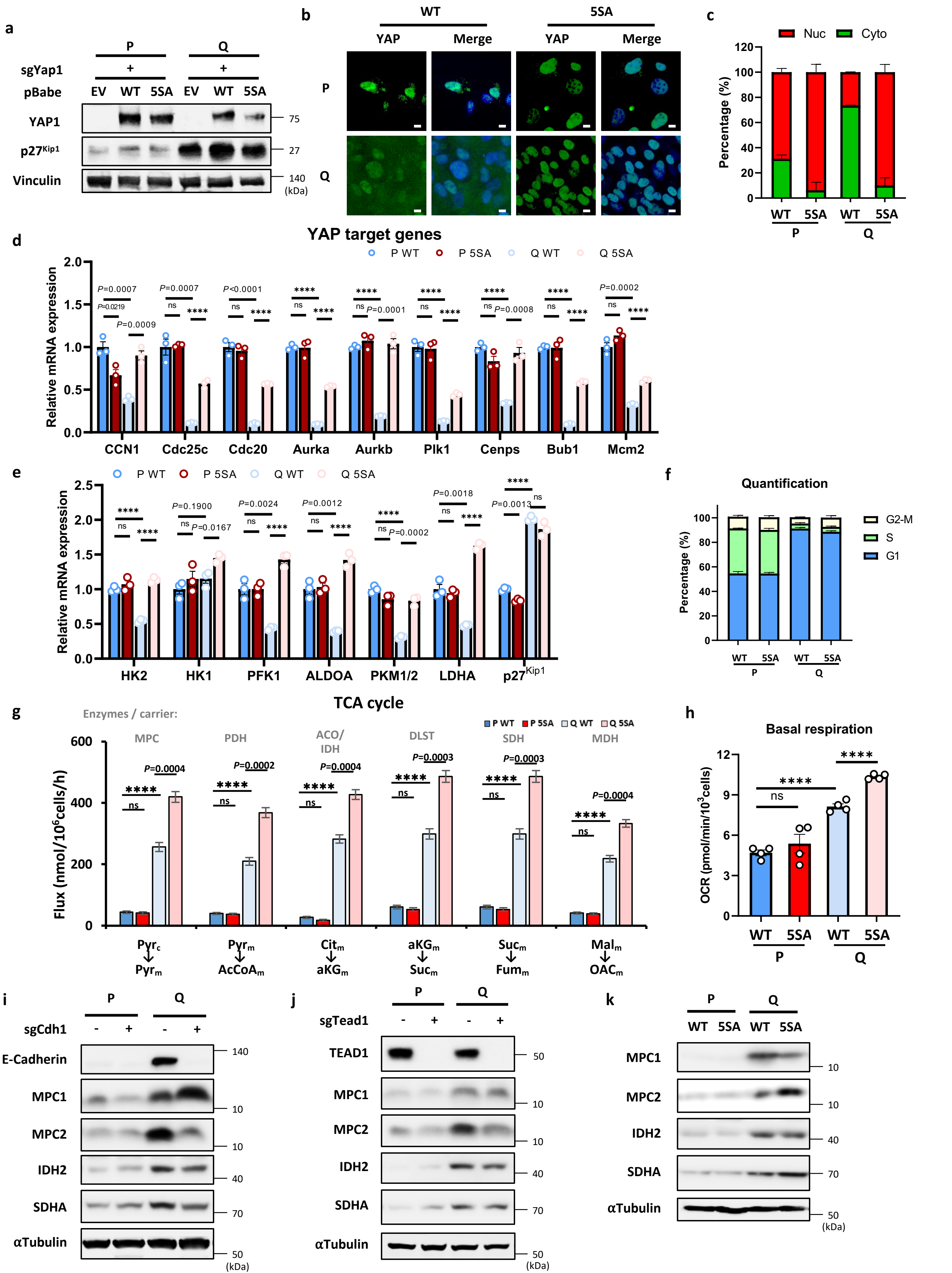

a
P
Q
sgYap1
+
+
pBabe
EV
WT
5SA
EV
WT
5SA
YAP1
75
p27Kip1
27
Vinculin
140
(kDa)
b
WT
5SA
YAP
Merge
YAP
Merge
P
Q
c
Nuc
Cyto
Percentage (%)
WT
5SA
WT
5SA
P
Q
d
YAP target genes
P WT
P 5SA
Q WT
Q 5SA
Relative mRNA expression
CCN1
Cdc25c
Cdc20
Aurka
Aurkb
Plk1
Cenps
Bub1
Mcm2
P=0.0007
P=0.0219
P=0.0009
P=0.0007
P<0.0001
P=0.0001
P=0.0008
P=0.0002
e
P WT
P 5SA
Q WT
Q 5SA
Relative mRNA expression
HK2
HK1
PFK1
ALDOA
PKM1/2
LDHA
p27Kip1
P=0.1900
P=0.0167
P=0.0024
P=0.0012
P=0.0002
P=0.0018
P=0.0013
f
Quantification
G2-M
S
G1
Percentage (%)
WT
5SA
WT
5SA
P
Q
g
TCA cycle
Enzymes / carrier:
MPC
PDH
ACO/ IDH
DLST
SDH
MDH
P WT
P 5SA
Q WT
Q 5SA
Flux (nmol/10^6cells/h)
P=0.0004
P=0.0002
P=0.0004
P=0.0003
P=0.0003
P=0.0004
Pyrc → Pyrm
Pyrm → AcCoAm
Citm → aKGm
aKGm → Sucm
Sucm → Fumm
Malm → OACm
h
Basal respiration
OCR (pmol/min/10^3cells)
WT
5SA
WT
5SA
P
Q
i
P
Q
sgCdh1
E-Cadherin
140
MPC1
10
MPC2
10
IDH2
40
SDHA
70
αTubulin
50
(kDa)
j
P
Q
sgTead1
TEAD1
50
MPC1
10
MPC2
10
IDH2
40
SDHA
70
αTubulin
50
(kDa)
k
P
Q
WT
5SA
WT
5SA
MPC1
10
MPC2
10
IDH2
40
SDHA
70
αTubulin
50
(kDa)

Supplementary figure 7

a. Immunoblots of YAP1, in P and Q MEFs after CRISPR-Cas9 based YAP1 deletion and either EV or YAP WT or YAP 5SA were re-expressed. Vinculin was monitored as a loading control. $p27^{Kip1}$ is monitored as a quiescent marker. The immunoblots are representative of three independent experiments.

b. Immunofluorescence analysis of YAP (green) in YAP WT (WT) and YAP 5SA (5SA) cells in P and Q states. Hoechst 33342 (blue) was used to show the nuclei, and the scale bar size represents 10 µm. Each experiment included observation of at least 10 randomly selected fields (400× magnification).

c. The quantified percentage (%) of nuclear (red) and cytosolic (green) YAP immunofluorescence is indicated, where the data represent the mean ± SEM (n=6~7). The statistical significance of the differences was determined by two-way ANOVA; *P* <0.0001.

d. Relative mRNA expression of YAP target genes in WT and 5SA cells in the P and Q states. Data are normalized to the average value of P WT cells and are the mean ± SEM of 3 independent experiments.

e. Relative mRNA expression of glycolytic enzymes and $p27^{kip1}$ in WT and 5SA cells in the P and Q states. Vinculin was monitored as a loading control.

f. Cytometric analysis of BrdU incorporation and DNA content by PI staining in WT and 5SA cells in the P and Q states. The graph shows the quantification of cells in each phase of the cell cycle: cells arrested in G_1 phase (BrdU negative; 2N DNA content), in S phase (BrdU positive) and in G_2-M phase (BrdU negative; 4N DNA content). The statistical significance of the differences was determined using two-way ANOVA; *P* <0.0001.

g. Comparison of key intracellular TCA cycle fluxes (estimated flux ± SD) in WT and 5SA cells in the P and Q states.

h. Basal respiration measured by oxygen consumption rate (OCR) of WT and 5SA cells in the P and Q states. Values are the mean ± SEM of 4 independent experiments.

i. Immunoblots of E-cadherin, MPC1-2, and TCA cycle enzymes in control or E-cadherin KO MEFs in the P and Q states. αTubulin was used as a loading control. Each immunoblot is representative of three independent experiments. Abbreviations: MPC, mitochondrial pyruvate carrier; IDH, isocitrate dehydrogenase; DLST, dihydrolipoamide S-succinyltransferase; SDHA, succinate dehydrogenase A.

j. Immunoblots of TEAD1, MPC1-2, and TCA cycle enzymes in control or TEAD1 KO MEFs in the P and Q states. αTubulin was used as a loading control. Each immunoblot is representative of three independent experiments.

k. Immunoblots of MPC1-2 and TCA cycle enzymes in WT and 5SA cells in the P and Q states. αTubulin was used as a loading control. Each immunoblot is representative of three independent experiments.

Means and SEMs are shown, unless otherwise indicated. Individual *P* values by unpaired two-tailed *t*-test are indicated in each figure, unless otherwise indicated. Otherwise, asterisks indicate the *P* value; ns *P* ≥0.05, * *P* <0.05, ** *P* <0.01, *** *P* <0.001, **** *P* <0.0001.

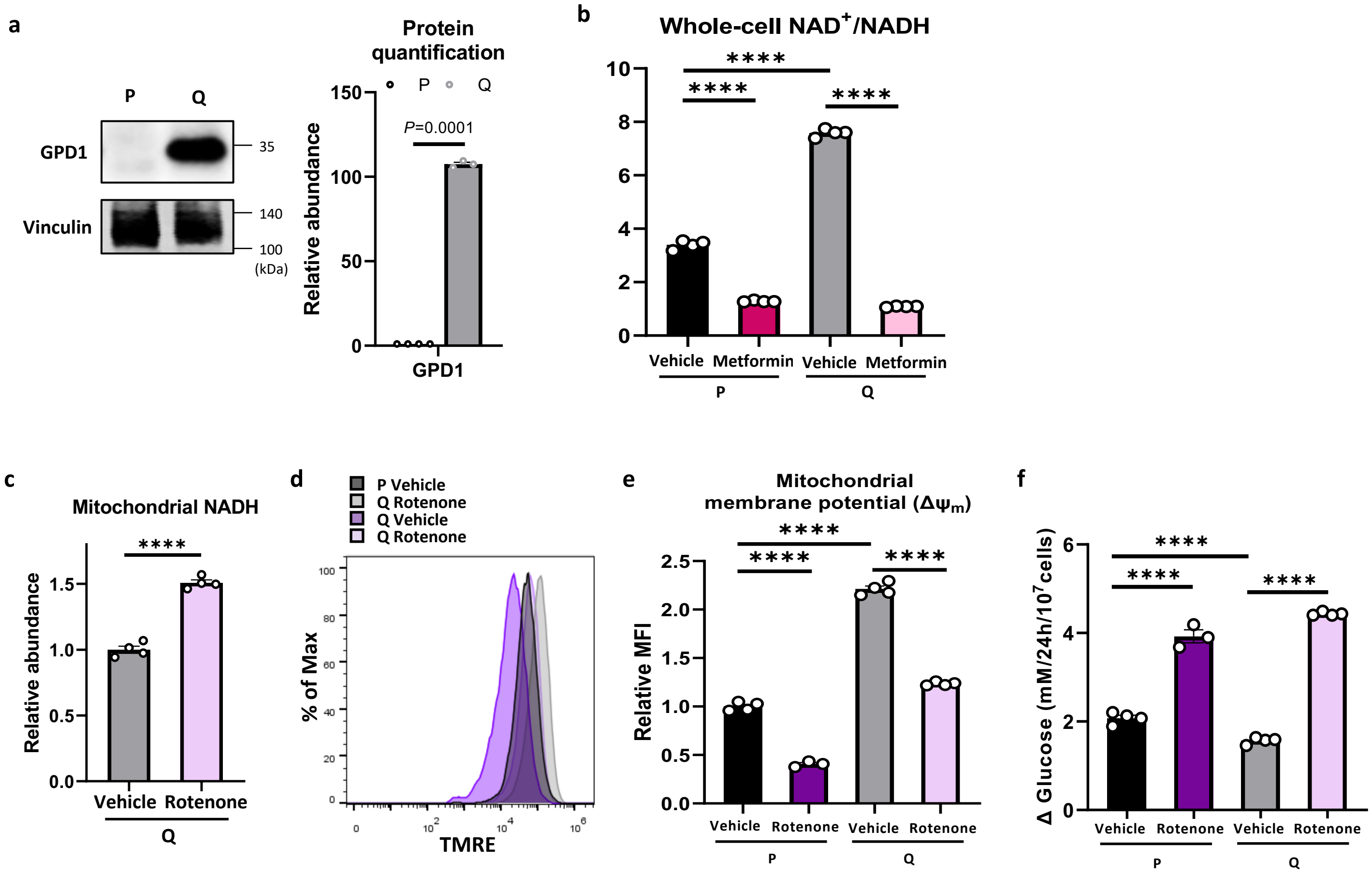

Supplementary figure 8

a. Immunoblot and protein quantification of glycerol-3-phosphate dehydrogenase 1 (GPD1) in P and Q MEFs. Vinculin was monitored as a loading control. Each immunoblot is representative of three independent experiments. Values are the mean ± SEM of 3 independent experiments.

b. Whole-cell NAD^+/NADH ratio in P or Q MEFs cultured in vehicle or 10 mM metformin for 24 h. Data are presented as the mean ± SEM of 4 biologically independent samples.

c. The relative abundance of mitochondrial NADH in Q MEFs cultured in vehicle or 0.2 µM rotenone for 24 h measured by the pC1-mitoRexYFP sensor using flow cytometry. Data are presented as the mean ± SEM of 4 biologically independent samples.

d,e. Mitochondrial membrane potential ($\Delta\Psi_m$), as reflected by TMRE fluorescence, of P and Q MEFs. Values are the mean ± SEM of 3 to 4 independent experiments.

f. Extracellular glucose uptake rates of P and Q MEFs cultured in vehicle or 0.2 µM rotenone for 24 h. The amount of extracellular glucose uptake in 24 h was normalized to the viable cell number. The results are the mean ± SEM of 3 to 4 independent experiments.

Means and SEMs are shown, unless otherwise indicated. Individual *P* values by unpaired two-tailed *t*-test are indicated in each figure, unless otherwise indicated. Otherwise, asterisks indicate the *P* value; ns *P* ≥0.05, * *P* <0.05, ** *P* <0.01, *** *P* <0.001, **** *P* <0.0001.

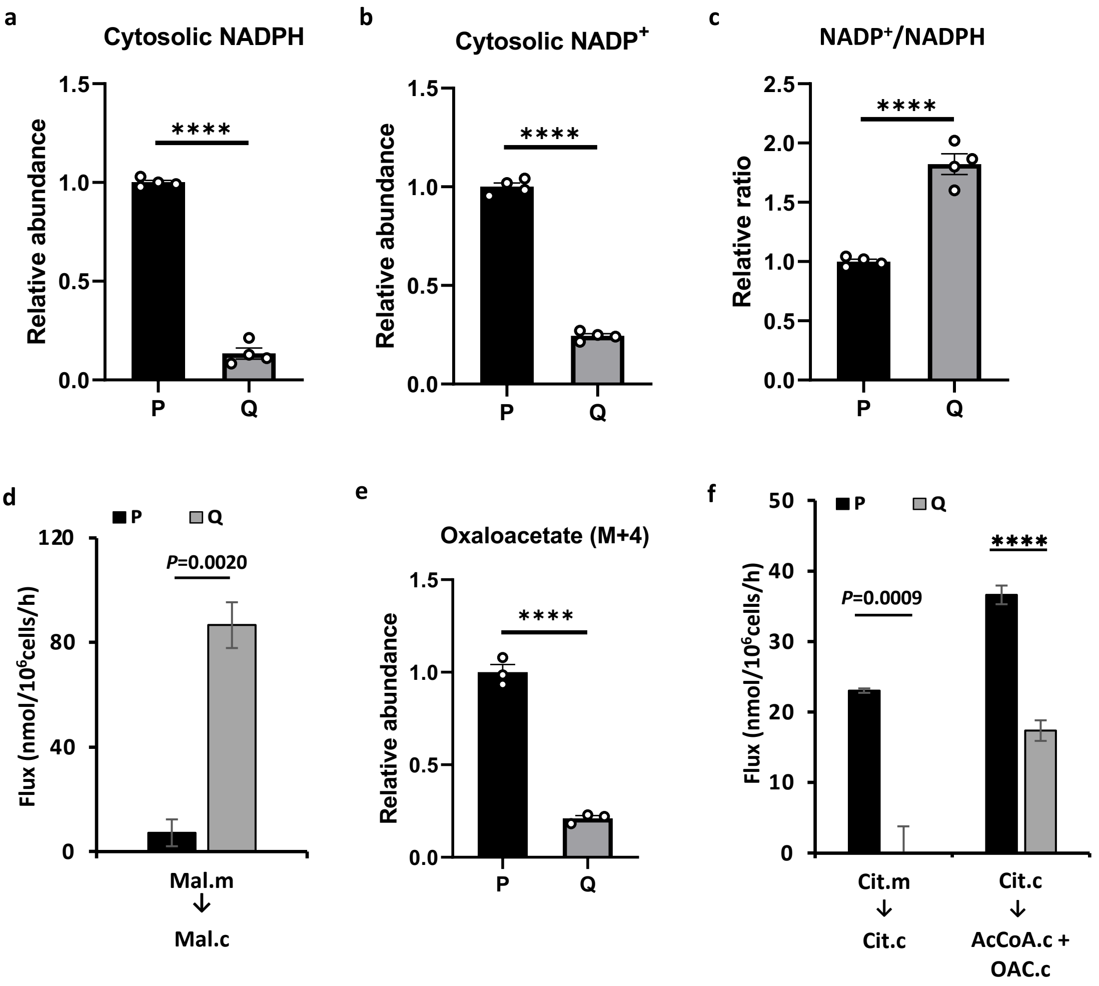

Supplementary figure 9

a. Relative abundance of cytosolic NADPH in P and Q MEFs measured by the iNap1 sensor (cytosolic) using flow cytometry. Data are presented as the mean ± SEM of 4 biologically independent samples.

b. Relative abundance of cytosolic $NADP^+$ in P and Q MEFs measured by the iNap1 sensor (cytosolic) using flow cytometry. Data are presented as the mean ± SEM of 4 biologically independent samples.

c. Relative ratio of cytosolic $NADP^+$/NADPH of P and Q MEFs measured by the iNap1 sensor (cytosolic) using flow cytometry. Data are presented as the mean ± SEM of 4 biologically independent samples.

d. Comparison of the quantified mitochondrial malate export flux (Mal.m → Mal.c) (estimated flux ± SD) in P and Q MEFs**.** P cells are shown in black bars, and Q cells are shown in gray bars.

e. Relative abundance of oxaloacetate (M+4) after 4 mM [U-^{13}C]-glutamine labeling for 24 h. The AUC was normalized to the levels of protein and internal standard. Means ± SEMs (n = 3) are shown.

f. Comparison of the quantified fluxes of mitochondrial citrate export (Cit.m → Cit.c) and conversion of cytosolic citrate (Cit.c) to cytosolic oxaloacetate (OAC.c) and acetyl-CoA (AcCoA.c) (estimated flux ± SD) in P and Q MEFs**.** P cells are shown in black bars, and Q cells are shown in gray bars.

Means and SEMs are shown, unless otherwise indicated. Individual *P* values by unpaired two-tailed *t*-test are indicated in each figure, unless otherwise indicated. Otherwise, asterisks indicate the *P* value; ns *P* ≥0.05, * *P* <0.05, ** *P* <0.01, *** *P* <0.001, **** *P* <0.0001.

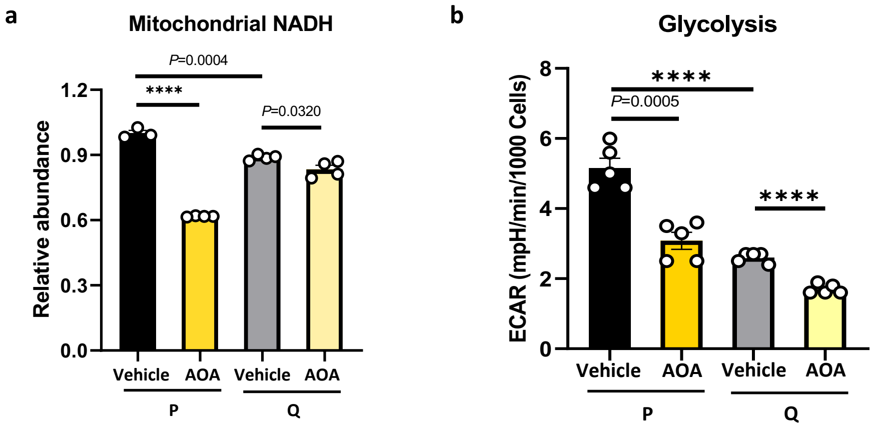

Supplementary figure 10

a. The relative mitochondrial NADH of P and Q MEFs cultured in vehicle or 2 mM AOA for 24 h measured by the pC1-mitoRexYFP sensor using flow cytometry. Values are the mean ± SEM of 3 to 4 biologically independent experiments.

b. Glycolysis measured by extracellular acidification rate (ECAR) of P and Q MEFs cultured in vehicle or 2 mM AOA for 24 h. Data are presented as the mean ± SEM of 5 biologically independent samples.

Means and SEMs are shown, unless otherwise indicated. Individual *P* values by unpaired two-tailed *t*-test are indicated in each figure, unless otherwise indicated. Otherwise, asterisks indicate the *P* value; ns *P* ≥0.05, * *P* <0.05, ** *P* <0.01, *** *P* <0.001, **** *P* <0.0001.

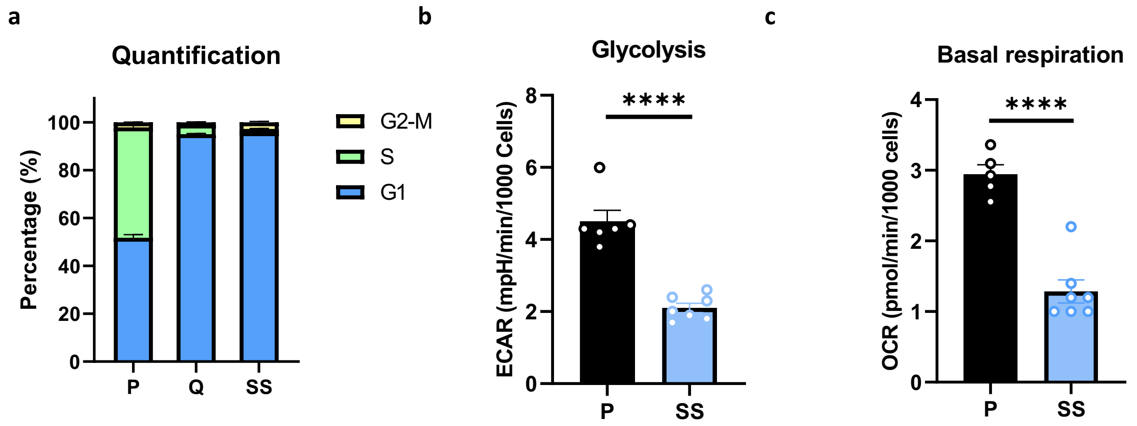

Supplementary figure 11

a. Quantification of the cytometric analysis of BrdU incorporation and DNA content by PI staining in P and serum-starvation induced SS MEFs. The graph shows the quantification of cells in each phase of the cell cycle: cells arrested in G_1 phase (BrdU negative; 2N DNA content), in S phase (BrdU positive) and in G_2-M phase (BrdU negative; 4N DNA content). The statistical significance of the differences was determined using two-way ANOVA; *P* <0.0001.

b. Glycolytic function monitored by the extracellular acidification rate (ECAR) in P and serum-starvation induced SS MEFs. Means ± SEMs (n ≥ 4) are shown.

c. Basal respiration measured by oxygen consumption rate (OCR) of P and serum-starvation induced SS MEFs. Means ± SEMs (n ≥ 4) are shown.

Means and SEMs are shown, unless otherwise indicated. Individual *P* values by unpaired two-tailed *t*-test are indicated in each figure, unless otherwise indicated. Otherwise, asterisks indicate the *P* value; ns *P* ≥0.05, * *P* <0.05, ** *P* <0.01, *** *P* <0.001, **** *P* <0.0001.
