# Supplementary material for: Metabolic and transcriptomic reprogramming during contact inhibition-induced quiescence is mediated by YAP-dependent and YAP-independent mechanisms
Source: Nat Commun. 2024 Aug 8;15:6777. doi: 10.1038/s41467-024-51117-y (PMC11310444; doi:10.1038/s41467-024-51117-y)
Supplement: Supplementary file 3 — Description of Additional Supplementary Files [file 41467_2024_51117_MOESM3_ESM.pdf]

Description of Additional Supplementary Files

Supplementary Data Legends

Supplementary Data 1 - Differentially Expressed Genes P versus Q cells

Supplementary Data 2 - 13C metabolic flux analysis results based on combined fitting of data from parallel labeling experiments with 1,2-13C

Supplementary Data 3 - Biomass specific uptake and production rates of extracellular metabolites (nmol10e6 cellsh n=8)
