# Supplementary material for: Metabolic and transcriptomic reprogramming during contact inhibition-induced quiescence is mediated by YAP-dependent and YAP-independent mechanisms
Source: Nat Commun. 2024 Aug 8;15:6777. doi: 10.1038/s41467-024-51117-y (PMC11310444; doi:10.1038/s41467-024-51117-y)
Supplement: Supplementary file 7 — Reporting Summary [file 41467_2024_51117_MOESM7_ESM.pdf]

Corresponding author(s): Hay, Nissim

Last updated by author(s): Jun 28, 2024

Reporting Summary

Nature Portfolio wishes to improve the reproducibility of the work that we publish. This form provides structure for consistency and transparency in reporting. For further information on Nature Portfolio policies, see our Editorial Policies and the Editorial Policy Checklist.

Statistics

For all statistical analyses, confirm that the following items are present in the figure legend, table legend, main text, or Methods section.

n/a	Confirmed	
☐	☒	The exact sample size (*n*) for each experimental group/condition, given as a discrete number and unit of measurement
☐	☒	A statement on whether measurements were taken from distinct samples or whether the same sample was measured repeatedly
☐	☒	The statistical test(s) used AND whether they are one- or two-sided<br>*Only common tests should be described solely by name; describe more complex techniques in the Methods section.*
☐	☒	A description of all covariates tested
☐	☒	A description of any assumptions or corrections, such as tests of normality and adjustment for multiple comparisons
☐	☒	A full description of the statistical parameters including central tendency (e.g. means) or other basic estimates (e.g. regression coefficient) AND variation (e.g. standard deviation) or associated estimates of uncertainty (e.g. confidence intervals)
☒	☐	For null hypothesis testing, the test statistic (e.g. *F*, *t*, *r*) with confidence intervals, effect sizes, degrees of freedom and *P* value noted<br>*Give P values as exact values whenever suitable.*
☒	☐	For Bayesian analysis, information on the choice of priors and Markov chain Monte Carlo settings
☒	☐	For hierarchical and complex designs, identification of the appropriate level for tests and full reporting of outcomes
☒	☐	Estimates of effect sizes (e.g. Cohen's *d*, Pearson's *r*), indicating how they were calculated

Our web collection on statistics for biologists contains articles on many of the points above.

Software and code

Policy information about availability of computer code

Data collection	Data collection (image acquisition and quantifications, metabolic analysis, and others) were performed with instruments and softwares as described in Methods.
Data analysis	Statistical analysis was performed with built-in statistics tools in Prism9 (GraphPad Software). Statistical significance was determined using Student's t test in paired groups of samples with known median and ordinary one-way analysis of variance (ANOVA) with Dunnett's or Holm–Sidak's multiple comparison test or two-way ANOVA with Tukey's post hoc multiple comparison test in comparisons of more than two groups of samples.

For manuscripts utilizing custom algorithms or software that are central to the research but not yet described in published literature, software must be made available to editors and reviewers. We strongly encourage code deposition in a community repository (e.g. GitHub). See the Nature Portfolio guidelines for submitting code & software for further information.

Data

Policy information about availability of data

All manuscripts must include a data availability statement. This statement should provide the following information, where applicable:

- Accession codes, unique identifiers, or web links for publicly available datasets
- A description of any restrictions on data availability
- For clinical datasets or third party data, please ensure that the statement adheres to our policy

Provide your data availability statement here.

Research involving human participants, their data, or biological material

Policy information about studies with human participants or human data. See also policy information about sex, gender (identity/presentation), and sexual orientation and race, ethnicity and racism.

Reporting on sex and gender	*Use the terms sex (biological attribute) and gender (shaped by social and cultural circumstances) carefully in order to avoid confusing both terms. Indicate if findings apply to only one sex or gender; describe whether sex and gender were considered in study design; whether sex and/or gender was determined based on self-reporting or assigned and methods used. Provide in the source data disaggregated sex and gender data, where this information has been collected, and if consent has been obtained for sharing of individual-level data; provide overall numbers in this Reporting Summary. Please state if this information has not been collected. Report sex- and gender-based analyses where performed, justify reasons for lack of sex- and gender-based analysis.*
Reporting on race, ethnicity, or other socially relevant groupings	*Please specify the socially constructed or socially relevant categorization variable(s) used in your manuscript and explain why they were used. Please note that such variables should not be used as proxies for other socially constructed/relevant variables (for example, race or ethnicity should not be used as a proxy for socioeconomic status). Provide clear definitions of the relevant terms used, how they were provided (by the participants/respondents, the researchers, or third parties), and the method(s) used to classify people into the different categories (e.g. self-report, census or administrative data, social media data, etc.) Please provide details about how you controlled for confounding variables in your analyses.*
Population characteristics	*Describe the covariate-relevant population characteristics of the human research participants (e.g. age, genotypic information, past and current diagnosis and treatment categories). If you filled out the behavioural & social sciences study design questions and have nothing to add here, write "See above."*
Recruitment	*Describe how participants were recruited. Outline any potential self-selection bias or other biases that may be present and how these are likely to impact results.*
Ethics oversight	*Identify the organization(s) that approved the study protocol.*

Note that full information on the approval of the study protocol must also be provided in the manuscript.

Field-specific reporting

Please select the one below that is the best fit for your research. If you are not sure, read the appropriate sections before making your selection.

☒ Life sciences ☐ Behavioural & social sciences ☐ Ecological, evolutionary & environmental sciences

For a reference copy of the document with all sections, see nature.com/documents/nr-reporting-summary-flat.pdf

Life sciences study design

All studies must disclose on these points even when the disclosure is negative.

Sample size	*Describe how sample size was determined, detailing any statistical methods used to predetermine sample size OR if no sample-size calculation was performed, describe how sample sizes were chosen and provide a rationale for why these sample sizes are sufficient.*
Data exclusions	No data exclusion.
Replication	WB quantifications were done with at least 3 indepedently generated blots.
Randomization	No randomization was required.
Blinding	No bilnding was required.

Reporting for specific materials, systems and methods

We require information from authors about some types of materials, experimental systems and methods used in many studies. Here, indicate whether each material, system or method listed is relevant to your study. If you are not sure if a list item applies to your research, read the appropriate section before selecting a response.

Materials & experimental systems

n/a	Involved in the study
☐	☒ Antibodies
☐	☒ Eukaryotic cell lines
☒	☐ Palaeontology and archaeology
☒	☐ Animals and other organisms
☒	☐ Clinical data
☒	☐ Dual use research of concern
☒	☐ Plants

Methods

n/a	Involved in the study
☒	☐ ChIP-seq
☐	☒ Flow cytometry
☒	☐ MRI-based neuroimaging

Antibodies

Antibodies used

MYC TAG Cell Signaling Technology 2276S
Flag TAG Cell Signaling Technology 2368S
Mouse monoclonal anti-FLAG Sigma-Aldrich F1804; RRID:AB_262044
HA TAG Sigma-Aldrich H-9658
Vinculin Sigma V9131; RRID:AB_477629
Tubulin Sigma 7-9062
HK1 Invitrogen MA5-15675
HK2 Cell Signaling Technology 2867S
PFK1 abcam ab97443
ALDOA Cell Signaling Technology 8060S
PKM1 Cell Signaling Technology 7067T
PKM2 R&D Systems MAB72442
LDHA Invitrogen MA5-17246
p27Kip1 BD Biosciences 610241
MPC1 Cell Signaling Technology 14462S
MPC2 Cell Signaling Technology 46141S
IDH2 Cell Signaling Technology 56439S
DLST Cell Signaling Technology 11954S
SDHA Cell Signaling Technology 11998S
Fumarase Cell Signaling Technology 4567T
CS Cell Signaling Technology 14309T
MDH2 Invitrogen PA5-21760
YAP1 Cell Signaling Technology 12395
Phosphorylated YAP1 (S127) Cell Signaling Technology 4911S
TEAD1 Cell Signaling Technology 12292S
PCK1 Cell Signaling Technology 12940S
MDH1 Invitrogen PA5-97965
ME1 Invitrogen MA5-23524
ME1 Proteintech 16619-1-AP
SIRT6 Invitrogen MA5-24768
ACAT1 Cell Signaling Technology 44276S
Acetylated Lysine Cell Signaling Technology 9441S
SLC25A11 Invitrogen PA5-101673
SLC25A13 Proteintech 10789-1-AP
GOT1 Proteintech 60317-1-Ig)
TOMM20 Invitrogen PA5-52843

GPD2 Proteintech 17219-1-AP
COL1A1 NOVUS BIO NBP1-30054
COL2A1 NOVUS BIO NBP1-77795
COL3A1 Proteintech 22734-1-AP
COL14A1 Invitrogen PA5-49916
COL23A1 Invitrogen MA5-24188
Goat anti-Mouse IgG (H+L) Cross-Adsorbed Secondary Antibody, Alexa Fluor™ 488 Invitrogen A11001
Goat anti-Rabbit IgG (H+L) Cross-Adsorbed Secondary Antibody, Cyanine3 Invitrogen A10520

Validation

All antibodies used herein are commercially available and have been validated by the companies that provide them.

Eukaryotic cell lines

Policy information about cell lines and Sex and Gender in Research

Cell line source(s)	ATCC , received from another source or established in the laboratory.
Authentication	All cell lines were received from ATCC and are NOT on the latest version (Vs. 8.0) of ICLAC list of misidentified cell lines. All lines used were tested for mycoplasma contamination and confirmed negative of infection via PCR through the UIC core.
Mycoplasma contamination	Cell lines used were tested for mycoplasma contamination and confirmed negative of infection via PCR through the UIC core.
Commonly misidentified lines (See ICLAC register)	Not relevant.

Plants

Seed stocks	*Report on the source of all seed stocks or other plant material used. If applicable, state the seed stock centre and catalogue number. If plant specimens were collected from the field, describe the collection location, date and sampling procedures.*
Novel plant genotypes	*Describe the methods by which all novel plant genotypes were produced. This includes those generated by transgenic approaches, gene editing, chemical/radiation-based mutagenesis and hybridization. For transgenic lines, describe the transformation method, the number of independent lines analyzed and the generation upon which experiments were performed. For gene-edited lines, describe the editor used, the endogenous sequence targeted for editing, the targeting guide RNA sequence (if applicable) and how the editor was applied.*
Authentication	*Describe any authentication procedures for each seed stock used or novel genotype generated. Describe any experiments used to assess the effect of a mutation and, where applicable, how potential secondary effects (e.g. second site T-DNA insertions, mosiacism, off-target gene editing) were examined.*

Flow Cytometry

Plots

Confirm that:

☐ The axis labels state the marker and fluorochrome used (e.g. CD4-FITC).

☐ The axis scales are clearly visible. Include numbers along axes only for bottom left plot of group (a 'group' is an analysis of identical markers).

☐ All plots are contour plots with outliers or pseudocolor plots.

☐ A numerical value for number of cells or percentage (with statistics) is provided.

Methodology

Sample preparation	*Describe the sample preparation, detailing the biological source of the cells and any tissue processing steps used.*
Instrument	*Identify the instrument used for data collection, specifying make and model number.*
Software	*Describe the software used to collect and analyze the flow cytometry data. For custom code that has been deposited into a community repository, provide accession details.*
Cell population abundance	*Describe the abundance of the relevant cell populations within post-sort fractions, providing details on the purity of the samples and how it was determined.*
Gating strategy	*Describe the gating strategy used for all relevant experiments, specifying the preliminary FSC/SSC gates of the starting cell population, indicating where boundaries between "positive" and "negative" staining cell populations are defined.*

☐ Tick this box to confirm that a figure exemplifying the gating strategy is provided in the Supplementary Information.
